# The role of tissue-based biomarkers in isolated REM sleep behavior disorder: implications for early detection and prognostication

**DOI:** 10.1093/sleep/zsaf203

**Published:** 2025-07-18

**Authors:** Alexander J Baumgartner, Amy W Amara

**Affiliations:** Department of Neurology, University of Colorado Anschutz Medical Campus, 12631 E. 17th Ave. Mail stop B185, Aurora, CO 80045 United States; Department of Neurosurgery, University of Colorado Anschutz Medical Campus, 12631 E. 17th Ave. Mail stop B185, Aurora, CO 80045 United States; Department of Neurology, University of Colorado Anschutz Medical Campus, 12631 E. 17th Ave. Mail stop B185, Aurora, CO 80045 United States

Despite their prevalence, the diagnosis of Parkinson disease (PD) and related disorders remains challenging. Estimates of the accuracy of the clinical diagnosis of PD range from 73.8% for non-experts to 83.9% for experts [1], compared to the gold standard of pathological examination. This accuracy is even lower in the early stages of the disease – the accuracy of the initial diagnosis made by a movement disorders expert may be less than 80% [1–3]. The ability to accurately diagnose these conditions early in their presentation is increasingly important for clinical trial enrichment, particularly since 40-60% of dopaminergic neurons in the substantia nigra have degenerated by the time patients develop typical motor manifestations of PD [4] – a phenomenon which may partially explain the lack of successful disease modification trials in PD. [5–7] To this end, multiple recent attempts have been made to define PD and Lewy body disorders along a biological spectrum that captures not only clinical features, but also pathological and molecular aspects of these diseases [8, 9].

In many cases, the earliest clinical features of PD and related diseases are several so-called “prodromal” symptoms, which include hyposmia, constipation, anxiety, depression, and REM sleep behavior disorder (RBD) [10]. Among these, isolated RBD (iRBD, i.e., not due to medication effect or another condition) confers the highest likelihood of developing PD, dementia with Lewy bodies (DLB), or Multiple System Atrophy (MSA), with a risk that ranges from 33.5% at five years follow-up to 96.6% at 14 years [11, 12]. Despite this known risk, it remains difficult to predict precisely which patients may phenoconvert to motor-manifest PD, and when. Median latency from RBD symptom onset to neurodegenerative disease diagnosis is 12 – 14 years [10], though some individuals convert much sooner and, in rare cases, RBD may exist for up to 30 years before the onset of overt motor symptoms [13]. Thus, there is a clear need for the development of biomarkers to aid in the identification of patients at risk and the prediction of timing of phenoconversion.

In the current issue of SLEEP, Bruschi and colleagues provide a scoping review of available literature on the use of molecular biomarkers to accurately diagnosis iRBD and identify those at highest risk for conversion PD, DLB, or MSA [14]. Candidate biomarkers included in the review are misfolded α-synuclein, neurofilament light chain (NfL), dopa decarboxylase (DDC), glial fibrillary acidic protein (GFAP), albumin ratio in cerebrospinal fluid (CSF) and serum, and emerging peripheral proteins and microribonucleic acids (miRNAs). Of these, α-synuclein has thus far received the most attention as a biomarker for iRBD, given its increasing utilization as an in vivo marker of synuclein deposition in Lewy bodies, Lewy neurites, and glial cytoplasmic inclusions [15–17]. Recent studies demonstrate high prevalence of α-synuclein in iRBD patients in various tissues. For example, in the DeNovo Parkinson Cohort (DeNoPa), 94.6% of CSF samples from iRBD participants had a positive α-synuclein seed amplification assay (SAA), with 92.6% being positive at baseline – in some cases, less than one year from the onset of RBD symptoms – indicating this marker likely becomes positive very early in the disease course [18]. Real-time quaking-induced conversion (RT-QuIC) analysis of CSF has been shown to distinguish iRBD from controls with sensitivities between 75 and 100% and specificities of 97-98% [19, 20]. Longitudinal studies have begun to demonstrate the ability CSF RT-QuIC to risk-stratify iRBD patients regarding their likelihood of conversion to PD or DLB [21]. One goal is to establish less invasive measures of misfolded α-synuclein. In blood, α-synuclein measured from neuronally-derived extracellular vesicles (EVs) can distinguish iRBD patients from controls with a sensitivity of 87% and specificity of 82% [22]. Skin biopsy can provide a quick and minimally invasive assay for synuclein pathology: immunofluorescence staining for α-synuclein from skin samples has been shown to distinguish iRBD patients from controls with 78-82% sensitivity and 100% specificity, which is superior to skin-based synuclein-SAA [23, 24].

Although not studied as extensively, other molecular biomarkers may be useful as well. Blood levels of NfL have demonstrated the ability to predict iRBD phenoconversion: a threshold of 22.93 pg/mL was able to distinguish PD converters from non-converters with a sensitivity of 75% and specificity of 83% [25]. A separate study found that a threshold of 21.3 pg/mL distinguishes MSA converters from non-converters with 100% sensitivity and 94.3% specificity [26]. However, the same study did not find any difference in NfL levels between PD/DLB converters and non-converters, though this may be due to the study’s relatively short follow-up (less than three years) [26]. A single longitudinal study of iRBD patients demonstrated that an elevated CSF/serum albumin ratio confers an increased risk of phenoconversion to PD, but not DLB, over a mean follow-up period of 7.63 years [27]. Prodromal PD patients, particularly those with iRBD or hyposmia and abnormal dopamine transporter (DaT) imaging, seem to have elevated CSF DDC levels [28, 29], though longitudinal studies detailing the predictive value of DDC are lacking.

There are some potential caveats to the utilization of these biomarkers in the management of patients with iRBD. It should be noted that controversy exists as to whether α-synuclein is truly a causal mechanism of neurodegeneration, or rather some sort of epiphenomenon of multiple pathologic mechanisms [30]. In this scenario, it may instead be the futile goal of synuclein removal, rather than timeliness of the intervention, that explains unsuccessful disease-modification trials in PD. [6, 7, 30] Furthermore, many of the biomarkers reviewed by Bruschi et al. are neither universally found in PD and related diseases, nor are specific to these conditions. For example, in the Parkinson’s Progression Markers Initiative, CSF α-synuclein SAA was positive in 67.5% of *LRRK2* gene mutation carriers with PD (compared to 93.3% for sporadic PD), and in only 12.5% of female, normosmic *LRRK2* carriers [31]. Similarly, elevated plasma NfL levels predict conversion to Alzheimer’s disease in patients with mild cognitive impairment (MCI) [32], and plasma GFAP levels are strongly correlated with markers of amyloid beta levels in iRBD or MCI patients [33], suggesting that may reflect non-specific neurodegeneration or co-pathology. Finally, although iRBD confers a high risk of PD, it may represent a distinct phenotype of prodromal PD, which includes accelerated cognitive decline and distinct patterns of α-synuclein propagation [34, 35], and thus may not capture the full clinical spectrum of PD.

Despite these limitations, the use of tissue-based biomarkers in the clinical care of patients with iRBD offers several new and exciting opportunities for earlier diagnosis, prognostication, and potential monitoring of disease modification. For tissue-based biomarkers to be broadly adopted in clinical care, further longitudinal studies are needed to validate their diagnostic and prognostic value. Standardization of laboratory techniques will be critical. It may also be helpful to incorporate other biomarkers to aid in the identification of iRBD patients at risk for phenoconversion, including neuropsychological assessments, electrophysiology (during both wakefulness and sleep), imaging, olfactory and autonomic testing, among others [36, 37]. If these can be accomplished, there is a real possibility of successful disease modification for PD in the near future.
